# The association between headache presentation, normal examination and neuroimaging findings: a retrospective analysis of patients presenting to a tertiary referral centre

**DOI:** 10.4314/ahs.v22i4.31

**Published:** 2022-12

**Authors:** Sharania Moodley, Ahmed I Bhigjee

**Affiliations:** Department of Neurology, Nelson R Mandela School of Medicine, University of KwaZulu-Natal, Durban, South Africa

**Keywords:** Headache, normal clinical examination, neuroimaging, headache red flags

## Abstract

**Background:**

There is a high worldwide burden of headaches. Selection of patients with headaches for neuroimaging, in the absence of traditional red flags, is imperative in guiding further management.

**Objectives:**

Determine the yield of neuroimaging findings in patients with headache and normal examination; and potentially identifying additional red flags.

**Methods:**

A retrospective consecutive chart review of patients with a main complaint of headaches and normal clinical examination were assessed at a tertiary hospital, over a 10-year period.

**Results:**

Cohort consisted of 114 patients. Unexpected or normal variants found in 20.2% of patients (23/114) and 11.4% (13/114) required change in management. The absence of nausea and vomiting (p=0.009) and absence of sharp type headaches in unexpected or normal variants group (p=0.03) were statistically significant. There was a higher chance of an abnormal neuroimaging study in men and HIV seropositive patients.

**Conclusions:**

Decision to neuroimage should be determined on an individual basis (demographic factors, history of headache and examination) as normal examination cannot preclude patients from unexpected findings on neuroimaging. Headache with nausea and vomiting in isolation may be associated with normal neuroimaging reflecting primary type headaches. Findings support a lower threshold to neuroimage men and HIV seropositive patients with headaches despite normal clinical examination.

## Introduction

Headaches are a global human experience. There is a lifelong prevalence of 96%^1^ and estimated worldwide prevalence of 50% by the World Health Organisation (WHO).^2^ Family physicians, emergency physicians, and neurologists are regularly faced with the dilemma of when or if neuroimaging is warranted in patients with headaches and a normal clinical examination. Most headaches are primary headache disorders (nearly 98%) and a small percentage of secondary headaches are important to recognise as they may be life threatening without timeous intervention.^3^

In the Global Burden of Disease Study 2019 - headache disorders ranked 14^th^ among global causes of disability-adjusted life years (DALYs) for all ages and both sexes.^4^ In Africa, an adult population-based study determined a one-year prevalence of all headaches as 45% in Ethiopia, compared with 62% in Zambia.^5^

Practitioners often refer patients for neuroimaging due to fear of missing a serious underlying treatable cause, subsequent medico-legal repercussions, disability caused by headaches and resultant medication overuse. In England, migraine alone is responsible for an annual loss of 25 million days from work or school, and is also associated with an annual cost of about 17 billion dollars in the United States of America.^6^,^7^

The selective use of neuroimaging in primary headaches is not cost effective, and result in patient anxiety, radiation exposure or contrast related adverse effects, implications on future insurance applications, and possibility of false-positive results.^8^ Incidental findings can result in further unnecessary investigations, and these findings may not account for presenting symptoms.^9^ The prevalence of incidentalomas on magnetic resonance imaging (MRI) of the brain was found to be 22% in an umbrella review.^10^ Callaghan et al highlighted that neuroimaging was frequently ordered during outpatient headache visits and this contributed to almost 1 billion dollars in annual costs.^11^ Whilst the cost of imaging is often emphasized, the value of a negative scan should not be underestimated providing both patient and clinician reassurance.

Neuroimaging of all patients with headaches may practically impossible as South African state funded public sector caters for 80% of the population. As a result, it is overburdened compared to the private sector.^12^ A study in a tertiary hospital with 24-week elective MRI waiting periods determined that service expansion would be necessary to decrease the waiting period.^13^ Given the underfunded and constrained resources in the state facilities, it is therefore essential to know which category of patients to refer.

Headache disorders have been recently classified, in the third edition of the International Classification of Headache Disorders, into primary, secondary, painful cranial neuropathies, other facial pain and other headache disorders. 14 This classification aids in further management by way of aiding in diagnosis,^14^ and therefore management. Migraine and tension type headaches, are the most common type of headache disorder^15^ and Holle et al advocated that these patients do not require neuroimaging as these patients do not have a higher rate of relevant cerebral pathology when compared to the general population.^9^ The United Kingdom National Clinical Guidelines Centre advises the traditional method of diagnosing primary headaches does not require neuroimaging and therefore should be avoided, as it is unlikely to change management or reveal abnormalities.^8^ Obtaining a detailed history of the patient's symptoms and clinical examination are the most important aspects in diagnosing headaches and further classifying headache type.^16^

Callaghan et al highlighted the routine practice of neuroimaging patients with primary headaches.^17^ Fouche et al in the Western Cape, South Africa found the most inappropriately requested scans were CT brains and provides local evidence across disciplines for inappropriate brain imaging.^18^

Other rare causes of headaches with a normal examination include systemic malignancy with resultant neoplastic meningitis^19^ and chronic daily headaches in menopausal or perimenopausal patients.^20^ Some traditional indicators of headache red flags in guiding further referral include the modified mnemonic ‘SNNOOP 10’ (systemic illness, neurologic signs, onset pattern, older age, pattern change and neoplasm history; recent onset of new headache; positional headache; precipitated by sneezing, coughing, or exercise; papilledema; progressive headache and atypical presentations; pregnancy or puerperium; painful eye with autonomic features; posttraumatic onset of headache; pathology of the immune system such as HIV; painkiller overuse or new drug at onset of headache).^21^

Our study aimed to determine the correlation of neuroimaging findings in patients presenting with headaches and a normal examination, as there is a paucity of local data to guide practitioners on when to refer for neuroimaging.

## Research method and design

This study is a retrospective chart review of all patients assessed at a tertiary hospital with a main complaint of headaches and a normal clinical examination from January 2008 to January 2018. The study setting was the Department of Neurology at Inkosi Albert Luthuli Central Hospital (IALCH), an urban based tertiary referral centre for regional and district hospitals in Kwa-Zulu Natal province in South Africa.

**Figure 1:**
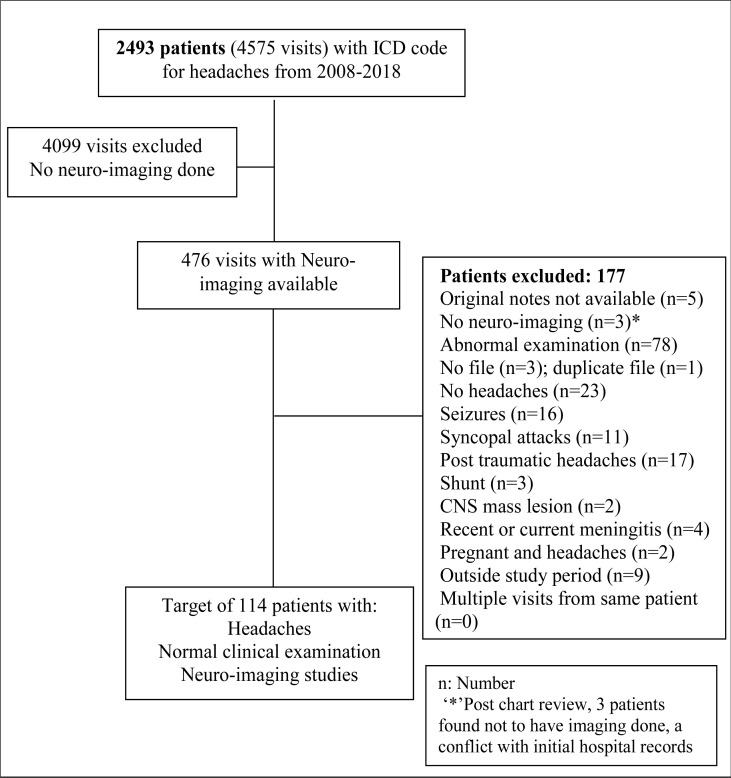
Flow chart of data collection

Patients with ICD coding for headaches were retrieved for the study period. (Figure 1)

Patients were included if they were 12 years and older with a main complaint of headaches, and a normal neurological examination with neuroimaging performed at IALCH.

Exclusion criteria included a history of cranial vault pathology and recent or current meningitis, headaches as a result of falls or trauma related injuries, post procedural headaches and pregnancy related headaches. The file with the most information was reviewed if duplicate files found. Patients were excluded if not assessed by a doctor from the neurology department.

The initial documented assessment at the neurology clinic was analysed. Neuroimaging (CT and MRI) studies were performed at the hospital (some patients may have undergone both studies) and these reports were reviewed. Data were collected for patient demographics (table 1) and headache characteristics (table 2). Neuroimaging findings were further evaluated as ‘normal’ or ‘unexpected findings and normal variants’ (table 3). This is because findings that were normal variants could not be deemed abnormal per se and all the other findings were classified as unexpected. This is in accordance with Kamtchum-Tatuene et al^20^. Human Immunodeficiency Virus (HIV) status was determined by disclosure by patient or testing at the hospital. If neither was done, the HIV status remained unknown.

**Table 1:** Patient demographics in all patients and subgroup analysis

Demographics	Subgroup analysis: neuroimaging		Whole cohort (n=114)	p value
	Normal (n=91)	Unexpected and normal variant (n=23)		
Age (years), n (%)				
12 to 30	34 (37.4)	5 (21.7)	39 (34.2)	0.09
31 to 40	23 (25.3)	3 (13.0)	26 (22.8)	
41 to 50	18 (19.8)	11 (47.8)	29 (25.4)	
>50	16 (17.6)	4 (17.4)	20 (17.5)	
				
Mean Age (mean, SD)	36.84 (14.10)	42.26 (15.88)	37.94 (14.57)	0.12
Male	34.66 (11.78)	35.78 (10.10)	34.92 (11.27)	0.81
Female	37.72 (14.93)	45.72 (17.56)	39.22 (15.65)	0.07
				
Gender, n (%)				
Male	26 (28.6)	8 (34.8)	34 (29.8)	0.56
Female	65 (71.4)	15 (65.2)	80 (70.2)	
				
Race, n (%)				
African	39 (42.9)	12 (52.2)	51 (44.7)	0.61
Indian	46 (50.5)	9 (39.1)	55 (48.2)	
Caucasian/ mixed	6 (6.6)	2 (8.7)	8 (7.0)	
				
Comorbidities*, n (%)				
Present	40 (44.0)	13 (56.5)	53 (46.5)	0.28
Absent	51 (56.0)	10 (43.5)	61 (53.5)	
				
HIV status, n (%)				
Seropositive	4 (4.4)	2 (8.7)	6 (5.3)	0.523
Seronegative	17 (18.7)	5 (21.7)	22 (19.3)	
Unknown	70 (76.9)	16 (69.6)	86 (75.4)	
				
CT brain, n (%)				
-Contrast	60 (67.4)	12 (66.7)	72 (67.3)	0.95
-Non-contrast	29 (32.6)	6 (33.3)	35 (32.7)	
-Not Applicable**			7	
				
MRI Brain, n (%)				
-Gadolinium	3 (50.0)	7 (77.8)	10 (66.7)	0.26
-Non-gadolinium	3 (50.0)	2 (22.2)	5 (33.3)	
-Not Applicable**			99	

* Comorbidities included: Hypertension, Diabetes Mellitus, Asthma, Ischaemic heart disease, Arthritis, Dyslipidaemia, Chronic kidney disease, Human Immunodeficiency virus, glaucoma, previous breast cancer, previous non-neurological related surgery, rheumatic heart disease, hyperthyroidism, Systemic lupus erythematosus, Major depressive disorder

** Not applicable in imaging modalities indicates that patients were imaged with the alternate imaging modality (7 patients did not undergo CT brain imaging and 99 patients did not undergo MRI brain imaging) Totals do not add up to 100 as figures rounded to one decimal point

**Table 2:** Headache characteristics in whole cohort and subgroup with normal versus unexpected findings or normal variant neuroimaging group

	Subgroup analysis: neuroimaging		Whole cohort (n=114)	p value
Headache characteristics	Normal (n=91)	Unexpected and normal variant (n=23)		
Start of headache, n (%)				
< 3months	27 (29.7)	3 (13.0)	30 (26.3)	0.67
3months-1 year	19 (20.9)	10 (43.5)	29 (25.4)	
> 1 year	37 (40.7)	8 (34.8)	45 (39.5)	
NA	8 (8.8)	2 (8.7)	10 (8.8)	
				
Headache location, n (%)				
Unilateral	21 (23.1)	5 (21.7)	26 (22.8)	0.33
Bilateral	17 (18.7)	4 (17.4)	21 (18.4)	
Holocephalic	14 (15.4)	3 (13.0)	17 (14.9)	
Localised region	37 (40.7)	8 (34.8)	45 (39.5)	
Unknown /not documented	2 (2.2)	3 (13.0)	5 (4.4)	
				
Onset, n (%)				
Sudden/acute/subacute	22 (24.2)	4 (17.4)	26 (22.8)	0.946
Chronic	5 (5.5)	1 (4.3)	6 (5.3)	
Gradual	8 (8.8)	2 (8.7)	10 (8.8)	
Not documented	56 (61.5)	16 (69.6)	72 (63.2)	
				
Frequency (per week)				
< 5	26 (28.6)	5 (21.7)	31 (27.2)	0.68
> 5	9 (9.9)	1 (4.3)	10 (8.8)	
Daily/alternate days	37 (40.7)	10 (43.5)	47 (41.2)	
Not documented	19 (20.9)	7 (30.4)	26 (22.8)	
				
Severity (pain-scale), n (%)				
Mild/moderate	4 (4.4)	1 (4.3)	5 (4.4)	0.052
Severe	39 (42.9)	4 (17.4)	43 (37.7)	
Not documented	48 (52.7)	18 (78.3)	66 (57.9)	
				
Duration, n (%)				
< 30 min	11 (12.1)	1 (4.3)	12 (10.5)	0.22
30 min-3 hrs	15 (16.5)	4 (17.4)	19 (16.7)	
3hrs - 7 days	28 (30.8)	3 (13.0)	31 (27.2)	
Constant	14 (15.4)	5 (21.7)	19 (16.7)	
Not documented	23 (25.3)	10 (43.5)	33 (28.9)	
				
Character, n (%)				
Sharp	17 (18.7)	0 (0.0)	17 (14.9)	0.03
Dull/pressure/other	21(23.1)	5 (21.7)	26 (22.8)	
Throbbing	32 (35.2)	7 (30.4)	39 (34.2)	
Not documented	21 (23.1)	11 (47.8)	32 (28.1)	
				
Constitutional symptoms, n (%)				
Present	6 (6.6)	1 (4.3)	7 (6.1)	0.88
Absent	76 (83.5)	21 (91.3)	97 (85.1)	
Not documented	9 (9.9)	1 (4.3)	10 (8.8)	
				
Nausea and vomiting, n (%)				
Present	36 (39.6)	4 (17.4)	40 (35.1)	0.009
Absent	54 (59.3)	16 (69.6)	70 (61.4)	
Not documented	1 (1.1)	3 (13.0)	4 (3.5)	
				
Visual disturbance, n (%)				
Present	16 (17.6)	4 (17.4)	20 (17.5)	0.30
Absent	75 (82.4)	18 (78.3)	93 (81.6)	
Not documented	0 (0.0)	1 (4.3)	1 (0.9)	
				
Photophobia and/or phonophobia, n (%)				
Present	33 (36.3)	9 (39.1)	42 (36.8)	0.81
Absent	58 (63.7)	14 (60.9)	72 (63.2)	
				
Other features, n (%)				
Present	22 (24.2)	8 (34.8)	30 (26.3)	0.33
Absent	63 (69.2)	14 (60.9)	77 (67.5)	
Not documented	6 (6.6)	1 (4.3)	7 (6.1)	
				
Autonomic features present, n (%)				
Yes	1 (1.1)	0 (0)	1(0.88)	0.90
No	91 (100)	23 (100.0)	113(99.12)	
				
Worse with Valsalva, n (%)				
Yes	11 (12.1)	3 (13.0)	14 (12.3)	0.71
No	66 (72.5)	15 (65.2)	81 (71.1)	
Not documented	14 (15.4)	5 (21.7)	19 (16.7)	
				
Medication response, n (%)				
No response	15 (16.5)	6 (26.1)	21 (18.4)	0.18
Good response	37 (40.7)	9 (39.1)	46 (40.4)	
No medication taken	4 (4.4)	3 (13.0)	7 (6.1)	
Not documented	35 (38.5)	5 (21.7)	40 (35.1)	

* Totals do not add up to 100 as figures rounded to one decimal point

n - number, HIV – Human Immunodeficiency Virus, min – minutes, hrs – hours.

**Table 3:** Unexpected and normal variants Neuroimaging findings

Unexpected and normal variants Neuroimaging findings	Number	Classification: Normal variant(NV) and unexpected findings (UF)	Change in management
Calcified granuloma	6	UF	No
Sinus disease	4	UF	Yes
Basal ganglia calcification	2	UF	No
Multiple rim-enhancing lesion	2	UF	Yes
Ischaemic leukoencephalopathy	1	UF	Yes
Basal ganglia infarct	1	UF	Yes
Vascular anomaly- pons nidus of vessels	1	UF	Yes
Atrophy of the parietal lobe	1	UF	No
Rathke cyst	1	UF	Yes
Meningioma	1	UF	Yes
Asymmetry of the lateral ventricles	1	NV	No
Supratentorial Hydrocephalus	1	UF	Yes
Enhancing rounded lesions are noted in left head of caudate nucleus and within the pons centrally	1	UF	Yes
Total	23	NV: 1 / UF: 22	No = 10, Yes = 13

The primary objectives were to determine the yield of neuroimaging findings in patients with normal clinical examination; and if the neuroimaging findings are clinically relevant. Secondary objectives aimed to identify additional red flags, if any, in patients with unexpected findings or normal variants and to estimate the cost to the state sector following further analysis of the scans.

Data collection was captured on Microsoft Excel 2010. Percentages were rounded off to the nearest decimal. To maintain anonymity, patients were identified by a unique number. Ethics approval was obtained from Biomedical Research Ethics Committee (BREC) and ethics consent was received on 18 July 2019. The ethics approval number is 134/19.

## Statistics

A sample of 114 patients presenting with headache and findings of a normal clinical examination is required to estimate the proportion of patients with abnormal neuroimaging findings to within ± 13% (37% - 63%) with probability of 95% and assuming an uninformed percentage of 50%. Sample size was estimated using Stata V13.1.

## Statistical analysis

Descriptive statistics on the demographic and clinical characteristics of patients is reported. Factors associated with abnormal neuroimaging (red flags) are identified using Chi Square tests for categorical variables and t test or Wilcoxon rank sum tests/Kruskal-Wallis for ordinal and numeric variables depending on their distribution. The effect of gender, age and HIV status is examined in a logistic model. Only unadjusted odds ratios and 95% confidence limits are reported since no variable reached the inclusion criteria of p < 0.3. Data was analysed using Stata Statistical software V15.1.

## Results

One hundred and fourteen consecutive patients with the main complaint of headache, normal neurological examination and neuroimaging available for analysis were retrospectively assessed (figure 1). Patients had a mean age of 37.9 years (range: 64; median 36) in the total cohort and 42.3 years (range 63; median 42.5) in the unexpected findings and normal variants neuroimaging group. The cohort was made up of mainly women (70.2%). Only 6 of 114 (5.3%) patients of cohort were HIV seropositive, 22 of 114 patients (19.3%) HIV seronegative and remainder unknown.

## Primary outcomes

Twenty-three of 114 patients (20.2%) were found to have unexpected or normal variant findings. Eleven of 23 patients (47.8%) were between 41–50 years of age and only 17.4% were older than 50 years of age. This did not reach statistical significance. Thirteen of 23 patients (56.5%) with unexpected and normal variants had neuroimaging findings that would require adjustment in management. The neuroimaging findings (table 3) were calcified granulomas (5.3%), sinus disease (3.5%) and one normal variant include (0.9%). The unexpected vascular findings were 2.6% (3 of 114%), neoplastic findings were 0.9% (1 of 114) and non-neoplastic findings were 15.8% (18 of 114).

## Secondary outcomes

Male patients were found to have a greater chance of having an unexpected finding or normal variant (table 4). More patients in the unexpected and normal variant group did not have nausea and vomiting and this reached statistical significance. (p = 0.009). The sharp type headaches were not present in the unexpected and normal variant group and also statistically significant (p = 0.03), however it was interpreted with caution as there were large numbers of missing data. There was no association with age in the two groups (p = 0.9). The chances of an unexpected finding or normal variant are almost twice as great in HIV seropositive compared to HIV seronegative patients but did not reach statistical significance (p = 0.60).

**Table 4:** Subgroup analysis of normal versus unexpected findings or normal variant neuroimaging group

	Subgroup analysis: Neuroimaging		Total	Chances having an unexpected or normal variant on neuroimaging			
	Normal (n=91)	Unexpected and normal variant (n=23)					
				p value	OR	95% CI	
Gender	n (%)	n (%)	n				
Men	26 (76.5)	8 (23.5)	34		ref	ref	ref
Women	65 (81.3)	15 (18.8)	80	0.56	0.75	0.28	1.98
							
Age							
<=50	75 (79.8)	19 (20.2)	94		ref	ref	ref
> 50	16 (80.0)	4 (20.0)	20	0.9	0.9	0.30	3.30
							
HIV status							
Seronegative	17 (77.3)	5 (22.7)	22		ref	ref	ref
Seropositive	4 (66.7)	2 (33.3)	6	0.60	1.70	0.24	12.17
Unknown	70 (81.4)	16 (18.6)	86	0.66	0.78	0.25	2.42

n - number, HIV – Human Immunodeficiency Virus

OR: odds ratio; CI: confidence interval, ref: reference

## Discussion

In the South African setting, the yield of neuroimaging findings in patients with a normal neurological has yet to be determined. This retrospective chart review revealed that patients in this category had unexpected or normal variants in 20.2%. This correlated with a study by Kamtchum-Tatuene et al, which revealed a prevalence of unexpected findings or normal variants on brain imaging to be 17.5% in patients with headaches and normal neurologic examination.^22^

Clinically significant abnormalities (defined as findings that would change management in patients) was found to be 11.4% (13 of 114) and is similar to a systematic review and meta-analysis by Jang et al which found an 8.86% prevalence of detecting clinically significant lesions in primary headache patients. (8) A prospective study by Sempere et al however detected significant intracranial abnormalities in 0.9% (95% CI 0.5, 1.4) in the same category of patients.^23^

In this cohort, patients with the highest frequency of unexpected findings or normal variants on neuroimaging were in the 41–50-year age range (47.8%) and only 17.4% were above the age of 50 years of age. This is in contrast to traditional red flags which include age over 50.^24^ This association did not reach statistical significance; however, these differences could be significant in a larger study.

The absence of nausea and vomiting between the two neuroimaging groups was found to be statistically significant. This is contrary to some studies which have recommended neuroimaging for headache aggravated by vomiting amongst other features.^25^ The patients with the normal neuroimaging had a higher percentage of nausea and vomiting 39.6% compared to 17.4% in the unexpected or normal variant group. Our study may largely reflect migraineurs reporting headaches associated with nausea and vomiting. This study further reinforces that the unexpected neuroimaging or normal variants can be present despite the absence of nausea and vomiting.

Two of the 6 HIV seropositive patients had unexpected findings or normal variants on neuroimaging. Although there were many patients whose status was unknown, the chances of having abnormal imaging are almost twice as great in HIV seropositive compared to HIV seronegative patients but did not reach statistical significance. This correlates with studies that deem HIV a red flag as it may reflect an immunosuppressed state.^26^

There was one patient with features of trigeminal autonomic cephalalgia (TAC) with normal neuroimaging. Guidelines indicate patients with TAC or migraine with a change in aura should be referred for MRI brain as initial imaging. This is because there is an unexplained association with pituitary macroadenomas in 4% of TAC patients^27^ and migraine with a change in aura may reflect occipital lesion.^28^ In this study, the presence of visual disturbance was not statistically significant and did not differentiate the presence of, type of or change in aura.

Kenteu et al highlighted that overuse of neuroimaging may result in frequent discovery of normal variants (NV) and incidental findings (IF) which most often do not explain the patient's pain.^29^,^30^ Our study found 1 normal variant. The other findings could not be classified as incidental as patients were symptomatic with headaches, and a description ‘unexpected findings’ is more appropriate. The incidence of subarachnoid haemorrhage (SAH) in patients with sudden severe headache and a normal neurological examination may be as high as 10%.^31^ Patients with acute onset headaches, elevated blood pressures, neck stiffness and altered mental state may also prompt further referral. Severity of headaches in our study approached statistical significance (p = 0.052) however the number of patients with missing data in this category was too high to place any relevance. Cerebral venous thrombosis (CVT) may be a life-threatening entity and present with thunderclap headache and should be further investigated with a CT brain. However, CT brain may be normal and an MRI or MR venogram should be performed if clinical suspicion persists.^32^ Our cohort did not have any patients with SAH or CVT.

The risk of a brain tumour increases with age and the presentation with an isolated headache can range between 2% and 16%.^33^ Our study revealed one intracranial meningioma in a 47-year-old female, 0.87% of the whole cohort. A study by Carey et al revealed the diagnosis of malignancy was rare in individuals presenting with incident headache and early neuroimaging (within 30 days of headache) led to a small reduction in time to diagnosis. 34 Interestingly, risk of death was higher in the early neuroimaging group compared to the referent group, and the authors postulate higher disease severity in this group.^34^ Therefore, timing of neuroimaging did not change outcomes.

The American College of Radiology (ACR) Appropriateness Criteria—Headache Clinical Variants (revised 2019) provides recent evidence-based guidelines^24^ and advise that initial imaging is usually not appropriate for patients with new primary migraine or tension-type headache with normal neurologic examination, or chronic headache with no new features. However, guidelines for neuroimaging in headaches (2019) by the British Society of Neuroradiologists Standards Subcommittee advise neuroimaging may be considered if a patient is disabled by fear of serious pathology. In our study, 114 patients were imaged despite the ACR criteria. A normal clinical examination correlated with 79.8% of normal neuroimaging. For the remaining 23 patients (20.2%), 1 patient had a normal variant and 22 had unexpected findings with 13 patients requiring change in management. The clinician is therefore guided largely by history (headache onset, progression and presence of red flags) as normal examination cannot rule out pathology completely when deciding on referral and neuroimaging.

The hospital management provided cost figures of MRI brain scans in 2008 (R4 096) and 2018 (R8 089), and cost of CT brain scans in 2008 (R1 628) and 2018 (R3 558), offering a range. One may consider that a normal scan (a minimum of 91 scans in this study) may have been avoided in such a population and thus a saving to both hospital and patient.

Strengths of our study included data collected from an electronic database. Data collection was done by one author and reduced the interpretation bias. Neuroimaging was done at a single location and this ensured uniformity in reporting and image acquisition protocols. Clinical assessments for this study were only considered from the neurology clinic, again ensuring consistency in history taking and clinical examination.

Limitations include retrospective design, and as a result missing data and risk of bias. There is potential for human error in allocating International Classification of Diseases (ICD) code in hospital systems. This study offers insights into neuroimaging in patients with a normal neurological examination at a single centre, however generalizability is limited. The study is also subject to referral bias, as it was conducted at a tertiary referral centre. This can overestimate or underestimate the rate of intracranial abnormalities.

To our knowledge, this is the first study of this nature in South Africa to correlate the neuroimaging findings of patients with headaches and normal clinical examination. Further prospective studies are recommended to assess adverse effects from neuroimaging and sensitivity and specificity of red flags in our setting.

Patients should be included in the decision-making process and counselled with regards to the benefits, harm and timing of neuroimaging. Defensive medicine may be reduced if clinicians are shielded by law when practicing evidence-based medicine in accordance with published guidelines.^35^ The practitioner plays an important role in the initial clinical assessment as serious illness can be detected despite normal imaging.^36^ Further, a normal investigation does not eliminate the need for further follow up and appropriate management of headache.

## Conclusion

We advise a lower threshold to refer patients with headaches and normal examination for neuroimaging if they are male, HIV seropositive, within the 41–50 year age group or experience a change in headache frequency and intensity. Importantly, this study demonstrates that headaches may still be associated with unexpected findings or normal variants on neuroimaging despite the absence of nausea and vomiting. In patients with headaches and a normal neurological examination, we advise referral of a subgroup of patients with primary headache disorders (trigeminal autonomic cephalalgias and migraine with change in aura).

## Data Availability

The data that support the findings of this study are available on request from the corresponding author, S.M. The data are not publicly available as it may compromise the privacy of research patients.
